# Edaravone Modulates Neuronal GPX4/ACSL4/5-LOX to Promote Recovery After Spinal Cord Injury

**DOI:** 10.3389/fcell.2022.849854

**Published:** 2022-05-18

**Authors:** Yilin Pang, Xinjie Liu, Xu Wang, Xuelian Shi, Lei Ma, Yan Zhang, Tiangang Zhou, Chenxi Zhao, Xu Zhang, Baoyou Fan, Jian Hao, Wenxiang Li, Xiaoqing Zhao, Rong Zhang, Songlin Zhou, Xiaohong Kong, Shiqing Feng, Xue Yao

**Affiliations:** ^1^ Tianjin Key Laboratory of Spine and Spinal Cord, International Science and Technology Cooperation Base of Spinal Cord Injury, Department of Orthopedics, International Chinese Musculoskeletal Research Society Collaborating Center for Spinal Cord Injury, Tianjin Medical University General Hospital, Tianjin, China; ^2^ Tianjin Key Laboratory of Metabolic Diseases, Department of Physiology and Pathophysiology, The Province and Ministry Co-sponsored Collaborative Innovation Center for Medical Epigenetics, Center for Cardiovascular Diseases, Research Center of Basic Medical Sciences, Tianjin Medical University, Tianjin, China; ^3^ Orthopedic Research Center of Shandong University, Cheeloo College of Medicine, Shandong University, Jinan, China; ^4^ Key Laboratory of Neuroregeneration of Jiangsu and Ministry of Education, Nantong University, Jiangsu, China

**Keywords:** edaravone, ferroptosis, neuroinflammation, neuroprotection, spinal cord injury

## Abstract

The FDA-approved drug edaravone has a neuroprotective effect on spinal cord injury (SCI) and many other central nervous system diseases. However, its molecular mechanism remains unclear. Since edaravone is a lipid peroxidation scavenger, we hypothesize that edaravone exerts its neuroprotective effect by inhibiting ferroptosis in SCI. Edaravone treatment after SCI upregulates glutathione peroxidase 4 (GPX4) and system Xc-light chain (xCT), which are anti-ferroptosis proteins. It downregulates pro-ferroptosis proteins Acyl-CoA synthetase long-chain family member 4 (ACSL4) and 5-lipoxygenase (5-LOX). The most significant changes in edaravone treatment occur in the acute phase, two days post injury. Edaravone modulates neuronal GPX4/ACSL4/5-LOX in the spinal segment below the lesion, which is critical for maintaining locomotion. Moreover, the GPX4/ACSL4/5-LOX in motor neuron is also modulated by edaravone in the spinal cord. Therefore, secondary injury below the lesion site is reversed by edaravone *via* ferroptosis inhibition. The cytokine array revealed that edaravone upregulated some anti-inflammatory cytokines such as IL-10, IL-13, and adiponectin. Edaravone reduced microgliosis and astrogliosis, indicating reduced neuroinflammation. Edaravone has a long-term effect on neuronal survival, spinal cord tissue sparing, and motor function recovery. In summary, we revealed a novel mechanism of edaravone in inhibiting neuronal ferroptosis in SCI. This mechanism may be generalizable to other neurological diseases.

## Highlights

Edaravone upregulates GPX4/xCT and downregulates ACSL4/5-LOX in the acute phase of SCI
Edarvone modulates neuronal GPX4/ACSL4/5-LOX in the spinal segment below the lesion, which is critical for maintaining locomotion
Edaravone reduces neuroinflammation and promotes long-term neuronal survival and functional recovery after SCI

## Introduction

“Time is spine” is a commonly accepted concept in traumatic spinal cord injury (SCI), indicating early intervention in the acute phase of SCI is very important (Bracken et al., 1997; Badhiwala et al., 2018; Courtine and Sofroniew, 2019; Huang et al., 2020). After SCI, there is a disruption in the microenvironment, and the intervention of cell death is the primary strategy of neuroprotection in the acute phase (Fan et al., 2018; Shi et al., 2021). We recently discovered that ferroptosis, a lipid peroxidation–induced programed cell death, plays an important role in SCI (Yao et al., 2019; Zhang et al., 2019). Recent research has revealed an emerging role for ferroptosis in the pathophysiology of acute CNS injuries such as stroke, traumatic SCI, and traumatic brain injury (Alim et al., 2019; Kenny et al., 2019; Hu et al., 2021). These CNS injuries shared similar mechanisms and neuroprotective agents. Because there is no effective drug approved in the clinic for the treatment of SCI, identifying ferroptosis inhibitors from FDA-approved drugs will meet the urgent need for medication for SCI.

Edaravone, also known as 3-methyl-1-phenyl-2-pyrazoline-5-one, is a free radical scavenger that was discovered in Japan in 2001 for the treatment of ischemic stroke and was later approved for the treatment of amyotrophic lateral sclerosis (ALS) in Japan (2015) and the United States (2017) (Rothstein, 2017). The use of edaravone in other disease models is rapidly expanding, from neurodegenerative diseases to inflammatory diseases (Jiao et al., 2015; Masuda et al., 2017; Reznik et al., 2020). Edaravone induces significant neuroprotection against SCI in rats by inhibiting lipid peroxidation (Ohta et al., 2005; Ohta et al., 2011; Ishii et al., 2018). Edaravone is intended to be a lipid peroxidation scavenger due to the lipophilic nature of phenyl and methyl groups, allowing edaravone to stay in the membrane and scavenge lipid ROS. Edaravone anion, an active form with antioxidant activity, donates an electron to free radicals, breaking the chain oxidation of lipids (Yamamoto et al., 1996). Because ferroptosis is caused by lipid peroxidation, edaravone’s anti-lipid peroxidation effect made it a potential ferroptosis inhibitor. Recently, edaravone has been reported to be a ferroptosis inhibitor in mouse hepatoma cell lines and a lipid peroxidation inhibitor (Homma et al., 2019). In addition, we discovered that edaravone inhibits ferroptosis in an oligodendrocyte cell line *in vitro* (Fan et al., 2021). Although edaravone has been shown to promote SCI repair in rodent models, the mechanism is unknown.

Here, we identify the role of edaravone as a ferroptosis inhibitor in contusion spinal cord injury in rats. Edaravone upregulates GPX4/xCT and downregulates ACSL4/5-LOX in the acute phase of SCI. Edaravone modulates neuronal GPX4/ACSL4/5-LOX in the spinal segment below the lesion, which is critical for maintaining locomotion. The mechanism we found on SCI may be generalizable to other CNS diseases.

## Materials and Methods

### Animals

Adult female Wistar rats (180 ± 20 g) were obtained from Beijing Vital River Laboratory Animal Technology Co., Ltd (Beijing, China, Permission Number: SCXK (Jing)—2016—0011) and housed for 1 week to acclimate to the environment and until their weight was up to 200–220 g. The experimental animal protocols were approved by the Ethics Committee of the Institute of Radiation Medicine, Chinese Academy of Medical Sciences (Tianjin, China, IRM-DWLI-2019-008) and were performed according to the National Institutes of Health Guidelines for the Care and Use of Laboratory Animals (7th Edition, National Academy Press, Washington DC).

### Spinal Cord Injury Surgery

All surgical procedures were carried out under aseptic conditions. The rats were initially anesthetized with 5% isoflurane (RWD life science, Shenzhen, China) and then maintained with 2–2.5% isoflurane (Venniro et al., 2018). A 1-cm midline incision was made over the thoracic vertebrae, and laminectomy on T10 and the caudal half of T9 vertebrae was performed. Spinal cord contusion injury was conducted by NYU Impactor Model III (W.M. Keck Center for Collaborative Neuroscience Rutgers, The State University of New Jersey, United States) using a 10-g node dropping freely from a height of 2.5 cm (Constantini and Young, 1994) and muscles and skin sutured in layers. Sham controls underwent laminectomy without the contusion. To prevent infection at the incision, cefuroxime sodium was applied for 3 days after injury. The bladders were emptied manually twice daily in the first week after injury.

### Drug Administration

The rats were randomly divided into three groups: the sham group, the SCI-vehicle group, and the SCI-EDV group. After SCI, the SCI-EDV group received edaravone (Sigma-Aldrich, MO, United States, 5 mg/kg). This dose was chosen according to a previous study on SCI rats (Ohta et al., 2005). Edaravone was dissolved in ethyl alcohol at 10 mg/ml and then diluted with sterile saline, with a final concentration of 1 mg/ml of edaravone and 10% ethyl alcohol. The rats were injected intraperitoneally with 5 mg/kg edaravone 30 min post injury and then once a day for 7 consecutive days after SCI.

### Western Blot

0.5-cm spinal cord tissues around the lesion center were harvested with PBS perfusion and homogenized on ice in RIPA lysis buffer (P0013B, Beyotime, Shanghai, China) to quantify ferroptosis-related proteins. 20 μg of the protein in each well was loaded on 12% SDS-PAGE and transferred onto a PDVF membrane, which was blocked with 5% skimmed milk at room temperature for 1.5 h followed by overnight incubation with primary antibodies against glutathione peroxidase 4 (GPX4, 1:5000, ab125066, and Abcam), System Xc^−^ light chain (xCT, 1:2000, ab175186, and Abcam), Acyl-CoA synthetase long-chain family member 4 (ACSL4, 1:5000, ab155282, and Abcam), 5-lipoxygenase (5-LOX, 1:1000, ab169755, and Abcam), and IBA-1 (1:1000, ab178846, and Abcam) at 4°C. After rinsing with Tris-buffered saline and Tween (TBST), the membrane was incubated with secondary antibody (Anti-mouse IgG, HRP-linked Antibody, 1:1000, 7076, CST; Anti-rabbit IgG, HRP-linked antibody, 1:1000, 7074, CST; Danvers, MA, United States) at room temperature for 1 h. Chemiluminescence was detected and quantified using ImageJ software. β-actin (1:5000, sc-47778, Santa Cruz Biotechnology, Santa Cruz, CA, United States) and GAPDH (1:1000, ab8245, and Abcam) were used as the internal standard. The mean and SD of relative protein levels across at least three independent experiments were used for statistical comparisons.

### Immunofluorescence Staining

The mice were transcardially perfused with PBS followed by perfusion with 4% paraformaldehyde, and tissues were fixed with 4% paraformaldehyde for 24 h. After being dehydrated by 30% sucrose, the spinal cord tissues were embedded in OCT and were cut at 10-μm thickness. The tissue sections were fixed with acetone for 30 min and then washed in phosphate-buffered saline (PBS). The sections were blocked with 5% goat serum at room temperature for 1 h and incubated overnight with the primary antibodies: glutathione peroxidase 4 (GPX4, 1:100, ab125066, Abcam, MA, United States), acyl-CoA synthetase long-chain family member 4 (ACSL4, 1:100, ab155282, Abcam, MA, United States), 5-lipoxygenase (5-LOX, 1:50, ab169755, Abcam, MA, United States), NeuN (1:200, ab104224, Abcam, MA, United States), ChAT (1:200,MA5-31382, Invitrogen, California, United States), CC-1 (1:200, OP80, Millipore, MA, United States), and GFAP (1:200, ab7260, Abcam, MA, United States) antibodies at 4°C, washed in phosphate-buffered saline with Tween (PBST), and incubated with secondary antibody (Goat Anti-Rabbit IgG H&L Alexa Fluor 488, 1:200, ab15008, and Goat Anti-Mouse IgG H&L Alexa Fluor 555), 1:200, ab150118; Abcam, MA, United States) for 1 h at room temperature. Immunofluorescent images were taken by a fluorescence microscope (Leica DMi8) (Figure 6) and confocal microscope (Figures 3, 4) (ZEISS LSM 900, Germany)/Vectra Polaris Automated Quantitative Imaging System (Vectra Polaris, America) (Figures 3, 4). The intensity was quantified by ImageJ software (NIH, Bethesda, MD, United States). All quantification was performed using ImageJ. Three images per animal were obtained from coronal spinal cord sections of the same epicenter and caudal position, and *n* = 3 animals in each group. The average mean density values are expressed as mean ± sd.

### Cytokine Array

According to the following method, the spinal cord epicenter tissues at 1 and 3 d post injury were collected and underwent a rat cytokine array (61 cytokines, G-series rat cytokine array, RayBiotech, Norcross, GA, United States). Chemiluminescence signals were detected using a ChemiDocMP System (Bio-Rad, Hercules, United States) and analyzed using ImageLab software (NIH, United States). The heat map was generated using TBtools (http://cj-chen.github.io/TBtools/) (Chen et al., 2020).

### Hematoxylin and Eosin (HE) Staining

HE staining was used to evaluate the injured spinal cord tissue after contusion. At 4 weeks post SCI, the rats were perfused intracardially with cold PBS and then with cold 4% paraformaldehyde. At the injury epicenter, a 1-cm spinal cord tissue was obtained and fixed for 24 h in 4% paraformaldehyde at 4°C. The sample was then embedded in paraffin and cut into 10-μm-thick cross-sections for HE staining, which was performed with a HE Staining kit (Beyotime, C0105, Shanghai, China) according to the manufacturer’s instructions.

### Locomotor Assessment

Basso, Beattie, and Bresnahan (BBB) scores are the hindlimb movement’s open-field locomotion test (Basso et al., 1995). BBB scores were evaluated weekly for up to 8 weeks. Briefly, rats in the open field were allowed to move freely for 5 min. Two independent blinded observers scored the rats on the hindlimb movement. For the objective assessment of locomotor coordination, the Catwalk gait analysis system (Noldus Information Technology B.V, Netherlands) was applied at 8 weeks after SCI as previously described (Hamers et al., 2006). Briefly, the rats were placed on the glass floor of the catwalk system in complete darkness, and all the rats were allowed to walk freely from left to right in the walkway. Paw prints were recorded by a high-speed video camera under the walkway. Catwalk software 10.6 was used to label each paw print during each run and analyze the gait parameters of the rats. The regularity index (%) is a common and comprehensive parameter to measure the degree of limb coordination.

### Neurological Functional Recovery Analysis

The rats were anesthetized at 8 weeks after injury with pentobarbital. Motor evoked potential (MEP) was detected at 8 weeks post SCI by electrophysiological devices (YRKJ-G2008; Zhuhai Yiruikeji Co, Ltd, Guangdong, China) after SCI as previously described (Yao et al., 2021). The backup skin of rats was disinfected after anesthesia, and the electrodes were placed. The reference electrodes were placed under the skin between the ears. The recording electrode was placed in the gastrocnemius muscle of the lower extremity, and the stimulation electrode was placed behind the head and neck. Single 5-mA stimulation was administered to stimulate the motor region of the cerebral cortex.

### Statistical Analysis

Statistical analysis was performed with GraphPad Prism (www.graphpad.com/scientific-software/prism/, San Diego, CA). *P* values < 0.05 were considered statistically significant. One-way ANOVA with Tukey’s *post hoc* test was used if more than two groups were compared and two-way ANOVA with Sidak’s multiple comparisons test if two independent variables were compared. Statistical details for specific experiments, including animal number, precision measures, statistical tests used, and definitions of significance, are described in figure legends.

## Results

### Edaravone Inhibits the Ferroptosis Pathway After SCI

**FIGURE 1:**
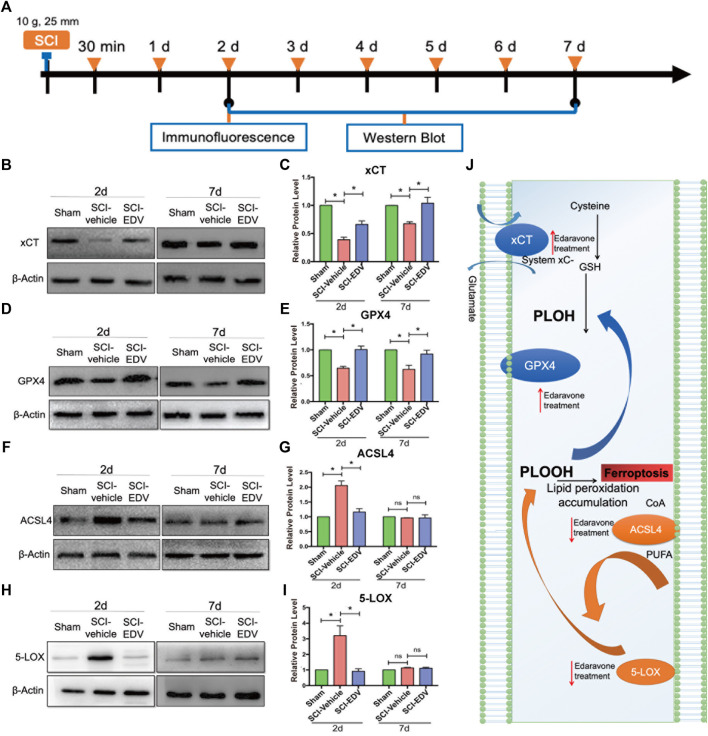
Edaravone modulates key proteins of the ferroptosis pathway in the spinal cord. **(A)** Schematic diagram of the experimental timeline. Wistar rats were intraperitoneally injected with 5 mg/kg edaravone daily during the first 7 days. At 2 and 7 days post SCI, tissues from spinal cord lesion epicenter were collected for western blot. At 2 days post injury the lesion epicenter and caudal spinal cord were observed from the immunofluorescence. **(B,D,F,H)** Representative western blot images of xCT **(B)**, GPX4 **(D)**, ACSL4 **(F)** and 5-LOX **(H)**. **(C,E,G,I)** Quantifications of three western blot band of xCT **(C)**, GPX4 **(E)**, ACSL4 **(G)** and 5-LOX **(I)**. Experiments were repeated three times. Data are expressed asmean ± standard deviation. Statistical significance was determined by one-way ANOVA with Tukey’s *post hoc* test. *p < 0.05, *n* = 3. **(J)** Diagram depicts that the ferroptosis pathway of SCI is modulated by edaravone.

We first investigate how the ferroptosis key proteins changed upon edaravone treatment in the spinal cord contusion model in rats. At 2 and 7 days after SCI, ferroptosis key proteins of the spinal cord lesion epicenter were detected by western blot (Figure 1A). Cystine/glutamate antiporter SLC7A11 (also commonly known as xCT) and Glutathione peroxidase 4 (GPX4), which are both ferroptosis suppressors (Koppula et al., 2021), are reduced in SCI-group at 2 and 7 days compared with the Sham group. The expression of xCT and GPX4 is rescued by edaravone treatment (*p* < 0.05, Figures 1B–E). Edaravone treatment in the sham group also showed elevated GPX4 level (Supplementary Figure S1). ACSL4, a biomarker to ferroptosis, (Verma et al., 2020) as well as 5-LOX, also pro-ferroptosis proteins, are both elevated at 2 days post injury, whereas edaravone downregulated the expression significantly (*p* < 0.05, Figures 1F–I). No significant change in the expression levels of 5-LOX and ACSL4 was detected at 7 days after SCI in any of the groups (*p* < 0.05, Figures 1F–I), indicating that ferroptosis mainly occurs in the acute phase. Immunofluorescence showed that GPX4 was abundant in the sham group but dramatically decreased in the SCI-vehicle group, whereas edaravone treatment rescued the GPX4 level (Supplementary Figure S2A). ACSL4 and 5-LOX increased after injury and decreased in the edaravone group (Supplementary Figure S2B,C). Altogether, the anti-ferroptosis regulators xCT and GPX4 were upregulated upon edaravone treatment, and the pro-ferroptosis regulators ACSL4 and 5-LOX were downregulated (Figure 1J), indicating the ferroptosis inhibition mechanism of edaravone.

### Edaravone Upregulates GPX4 After SCI

**FIGURE 2:**
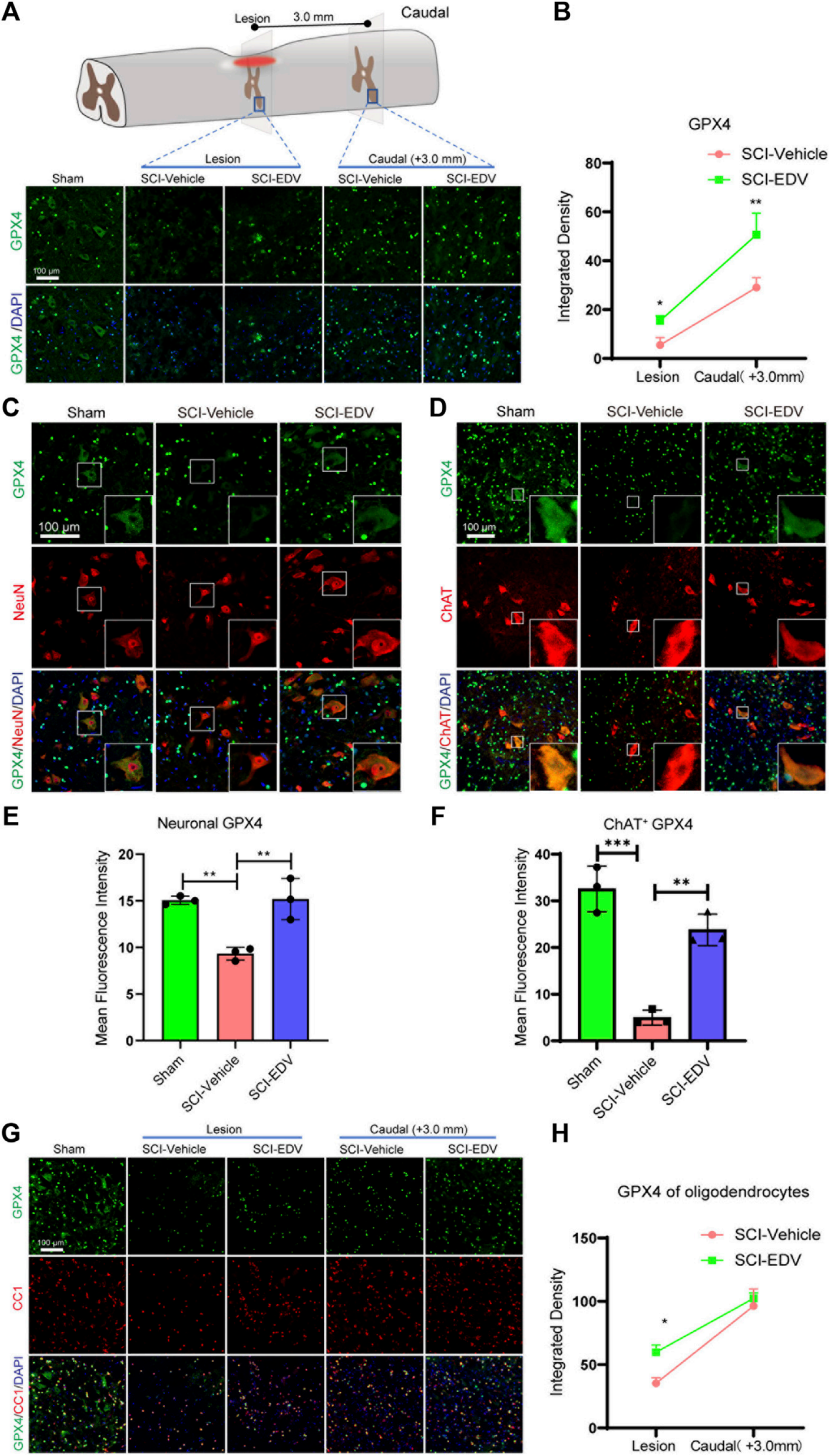
Edaravone upregulates GPX4 after SCI **(A)** Upper, schematic diagram of GPX4 detected injured epicenter and caudal spinal cord ventral horn (3 mm to the lesion) at 2 days post SCI, Lower, immunofluorescence images of GPX4 (green) in the lesion epicenter and caudal spinal cord section. Nuclei were stained with DAPI (blue). Scale bar, 100 μm. **(B)** Quantification of the GPX4 intensity in the lesion and caudal segment. **(C,D)** Representative immunofluorescence images of GPX4 (green)/NeuN (red) **(C)** and GPX4 (green)/ChAT (red) **(D)** in the caudal spinal cord at 3.0 mm to the lesion at 2 days post SCI. Scale bar,100 μm. **(E,F)** Quantification of Neuronal (NeuN positive) GPX4 **(E)** and Motor neuronal (ChAT positive) GPX4 **(F)**. **(G)** Representative immunofluorescence images of GPX4 (green)/CC1 (red) in both lesion epicenter and in the caudal spinal cord at 3.0 mm to the lesion at 2 days post SCI. Scale bar,100 μm. **(H)** Quantification of oligodendrocyte (CC1 positive) GPX4. Data are expressed as mean ± standard deviation. Statistical significance was determined by two-way ANOVA with Sidak’s multiple comparisons test **(B,H)** or one-way ANOVA with Tukey’s *post hoc* test **(E,F)**. *p < 0.05, **p < 0.01, and ***p < 0.001, *n* = 3, bar = 100 μm.

The lesion epicenter and caudal lumbar region represent the sites where the primary and secondary injury happens, respectively (Figure 2A). Total GPX4 decreased both in the lesion and caudal region after injury but increased after edaravone treatment (Figure 2B). Moreover, the spinal cord segment below the lesion is responsible for maintaining locomotion, and whether the neurons in lesion-adjacent spinal segments are influenced by the ferroptosis pathway is worth investigating. In the normal spinal cord, the GPX4 were expressed in two patterns, in the cytosol and in the nucleus. In colocalization with the neuronal marker NeuN, it expressed in the cytosol of the neurons. Edaravone rescued GPX4 levels in the neurons (Figures 2C,E). Edaravone rescued the GPX4 level in the motor neuron as well, shown by increased GPX4 in the ChAT-positive cell (Figures 2D,F). Double-labeling immunofluorescence showed that part of the GPX4 positive cells are CC1 positive, which represent mature oligodendrocytes. And this GPX4+CC1+ cells have a typical nucleus subcellular localization. However, the treatment increased oligodendrocyte GPX4 only in the epicenter but did not change in the caudal region after SCI (Figures 2G,H). Therefore, the most significant changes may occur in the neuronal level of GPX4 upon edaravone treatment.

### Edaravone Downregulates Neuronal ACSL4 and 5-LOX After SCI

**FIGURE 3:**
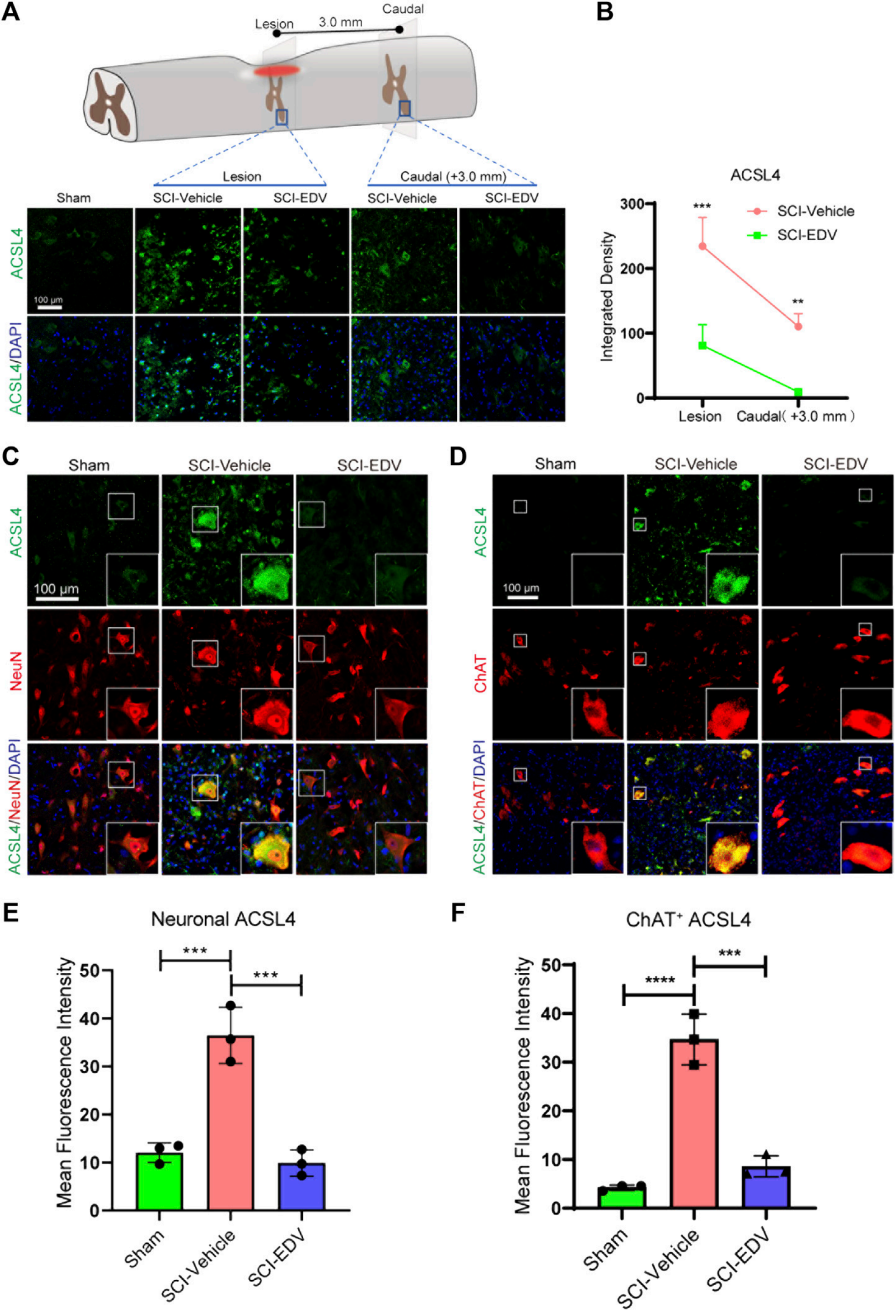
Edaravone downregulates ACSL4 at 2 days post SCI. **(A)** Upper, schematic diagram of ACSL4 detected injured epicenter and caudal spinal cord ventral horn (3 mm to the lesion) at 2 days post SCI. Lower, immunofluorescence images of ACSL4 (green) in the lesion epicenter and caudal spinal cord section. Nuclei were stained with DAPI (blue). Scale bar,100 μm. **(B)** Quantification of the ACSL4 intensity in the lesion and caudal segment. **(C,D)** Representative immunofluorescence images of of ACSL4 (green)/NeuN (red) **(C)** and ACSL4 (green)/ChAT (red) **(D)** in the caudal spinal cord at 3.0 mm to the lesion at 2 days post SCI. Scale bar,100 μm. **(E,F)** Quantification of neuronal (NeuN positive) ACSL4 **(E)** and motor neuronal (ChAT positive) ACSL4 **(F)**. Data are expressed as mean ± standard deviation. Statistical significance was determined by two-way ANOVA with Sidak’s multiple comparisons test **(B)** or one-way ANOVA with Tukey’s post hoc test **(E,F)**. *p < 0.05, **p < 0.01, and ***p < 0.001, *n* = 3, bar = 100 μm.

In the sham group, the ACSL4 level is very low, and total ACSL4 level in the SCI group increased both in the epicenter and lumbar segment below the lesion. In the lesioned spinal cord, the ACSL4 was expressed both in the cytosol and in the nucleus. Edaravone decreases the ACSL4 level in these two regions (Figures 3A,B). In the spinal cord segment below the lesion, NeuN-positive ACSL4 increased significantly in the injury group, whereas edaravone attenuated the ACSL4 level in neurons (Figures 3C,E). ChAT-positive cells (motor neurons) show a similar significant decrease in ACSL4 in the SCI-EDV group (Figures 3D,F).

**FIGURE 4:**
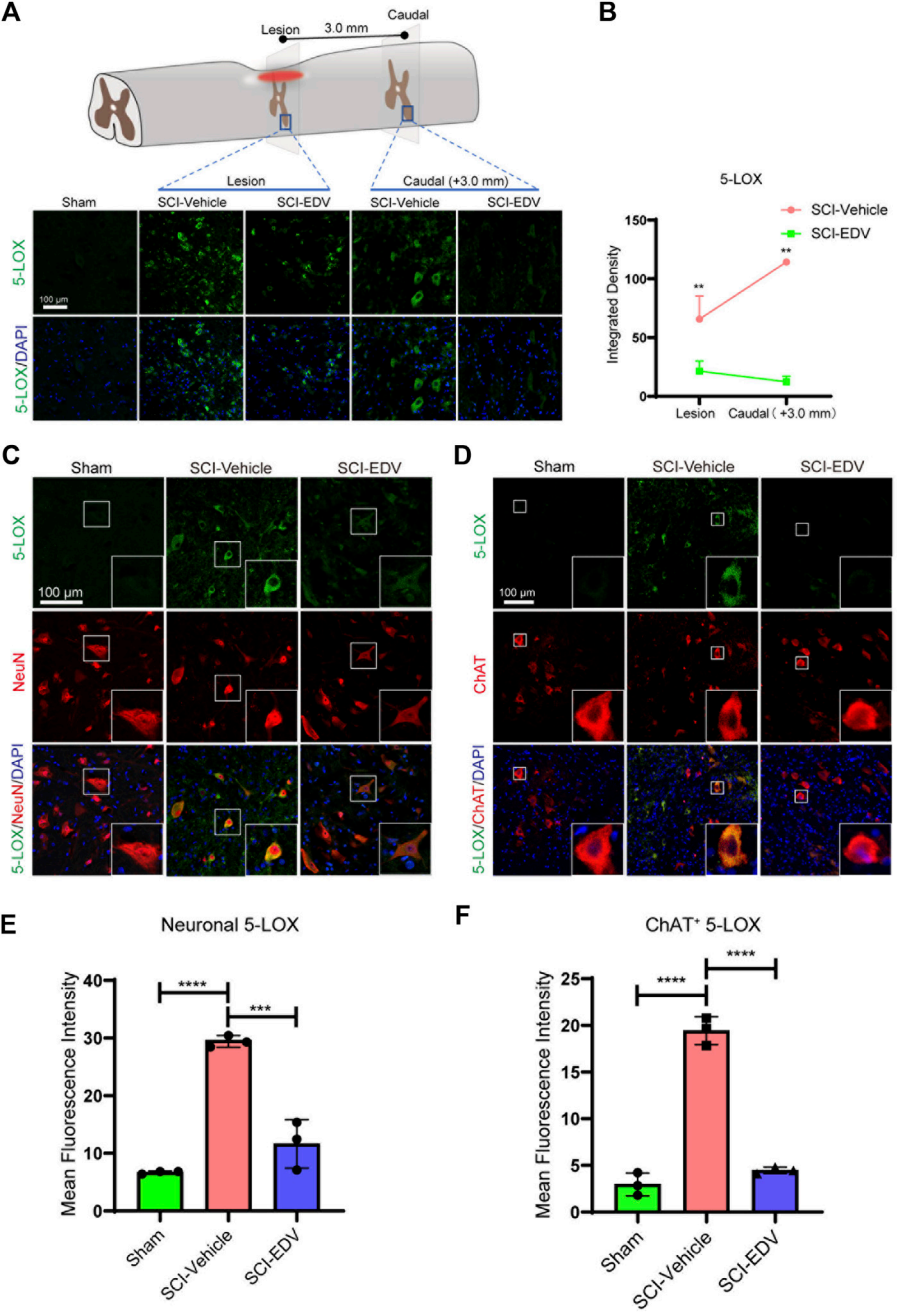
Edaravone downregulates 5-LOX at 2 days post SCI. **(A)** Upper, schematic diagram of 5-LOX detected injured epicenter and caudal spinal cord ventral horn (3 mm to the lesion) at 2 days post SCI, Lower, immunofluorescence images of 5-LOX (green) in the lesion epicenter and caudal spinal cord section. Nuclei were counterstained with DAPI (blue). Scale bar,100 μm. **(B)** Quantification of the 5-LOX intensity in the lesion and caudal segment. **(C,D)** Representative immunofluorescence images of 5-LOX (green)/NeuN (red) **(C)** and 5-LOX (green)/ChAT (red) **(D)** in the caudal spinal cord at 3.0 mm to the lesion at 2 days post SCI. Scale bar, 100 μm. **(E,F)** Quantification of neuronal (NeuN positive) 5-LOX **(E)** and motor neuronal (ChAT positive) 5-LOX **(F)**. Nuclei were stained using DAPI in blue. Data are expressed as mean ± standard deviation. Statistical significance was determined by two-way ANOVA with Sidak’s multiple comparisons test **(B)** or one-way ANOVA with Tukey’s *post hoc* test **(E,F)**. *p < 0.05, **p < 0.01, and***p < 0.001, *n* = 3, bar = 100 μm.

The total 5-LOX level increased both in epicenter and caudal region to the lesion after injury but decreased after edaravone treatment (Figures 4A,B). The change is more obvious in the caudal region. Neuronal colocolization with 5-LOX in the caudal region increased significantly in the injury group, whereas it was attenuated by edaravone (Figures 4C,E). ChAT-positive cells (motor neurons) show the same significant change (Figures 4D,F).

In summary, we demonstrate that the overall level of pro-ferroptosis proteins ACSL4 and 5-LOX was upregulated upon injury and downregulated after evaradone treatment. The neuron, especially the motor neuron, shows very significant downregulation of ACSL4 and 5-LOX upon edaravone treatment.

### Edaravone Attenuates Neuroinflammation After SCI

**FIGURE 5:**
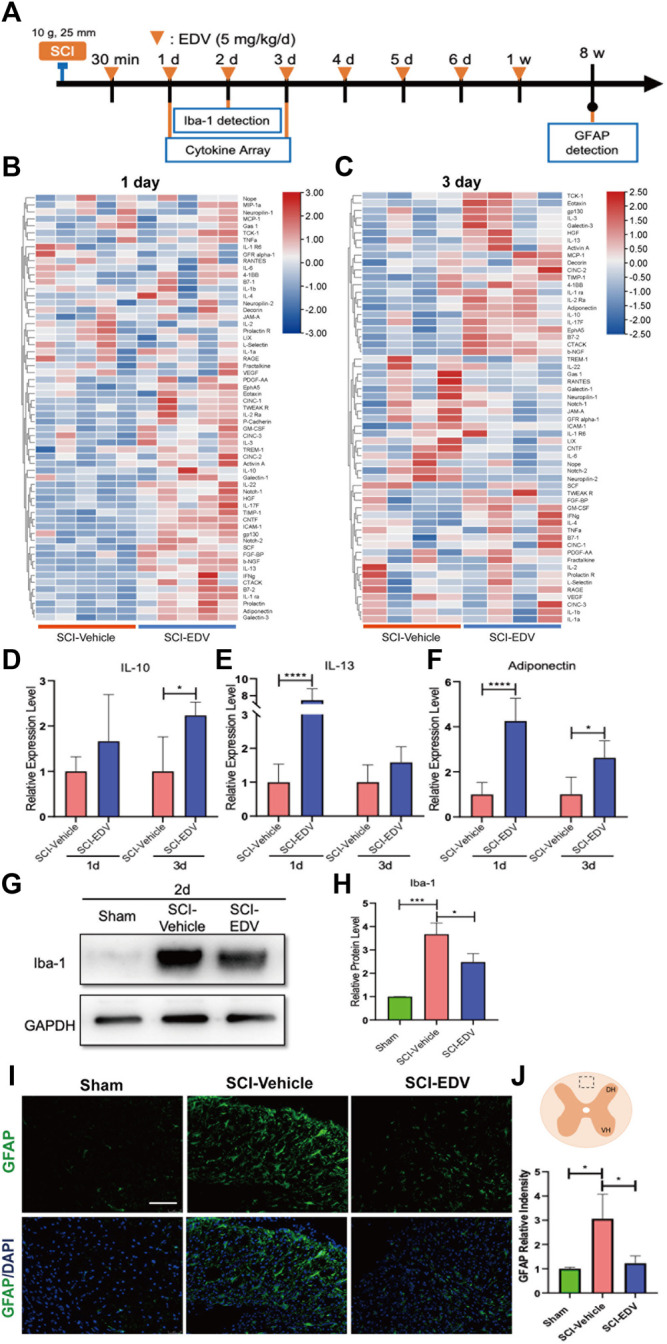
Edaravone reduced inflammation in the acute phase post SCI. **(A)** Timeline of the experiment about edaravone’s effect on inflammation. **(B,C)** Heatmap showing the changing proteins between SCI-Vehicle and SCI-EDV groups at 1d **(B)** and 3d **(C)** post SCI. **(D–F)** Quantifications of the relative levels of IL-10 **(D)**, IL-13 **(E)** and adiponectin **(F)**. Data are expressed as mean ± standard deviation. **(G,H)** Western blot analysis of Iba-1 **(G)** and quantification **(H)** at 2d post SCI are shown. **(I)** Spinal cord injury epicenter GFAP immunofluorescence 8 weeks post SCI. Scale bar = 100 μm. **(J)** Upper, the marked square in the spinal cord diagram indicates the field of microscope chosen for the representative images. Lower, quantification of GFAP. Statistical significance was determined by two-way ANOVA with Sidak’s multiple comparisons test **(D–F)** or one-way ANOVA with Tukey’s *post hoc* test **(H,J)**. *p < 0.05, ***p < 0.001, and ****p < 0.0001, *n* = 5 (cytokine array at 1 d. p.i.), *n* = 4 (3 d. p.i.), bar = 100 μm **(I)**.

Cytokine changes are also important aspects of the microenvironment after SCI. At 1 and 3 days post injury, spinal cord tissues were collected and analyzed by anti-cytokine antibody arrays containing antibodies to 61 inflammatory mediators (Figure 5A). The heatmaps of the SCI-vehicle and SCI-EDV groups at 1 d. p.i (Figure 5B) and 3 d. p.i. (Figure 5C). are shown. Among them, the expression of IL-10 was significantly upregulated at 3 days post injury after treatment of edaravone (Figure 5D). IL-13 was significantly higher at 1-day post SCI in the EDV treatment group compared with that in the SCI-vehicle group (Figure 5E). In addition, the level of adiponectin was upregulated both at 1 and 3 days after SCI in the edaravone group (Figure 5F).

Ionized calcium-binding adapter molecule 1 (Iba-1) is a critical marker of microglia. To further observe microglia activation after treatment by edaravone, the expressions of Iba-1 in the spinal cord were detected. The result showed that the expression of Iba-1 increased 2 days post injury, and edaravone treatment inhibits the expression of Iba-1 (Figures 5G,H). Excessive astrogliosis was observed in the SCI group when compared with the sham group. However, edaravone treatment significantly inhibited astrogliosis levels compared with those in the SCI group (Figures 5I,J).

### Edaravone Improves Long-Term Neuronal Survival and Tissue Sparing

**FIGURE 6:**
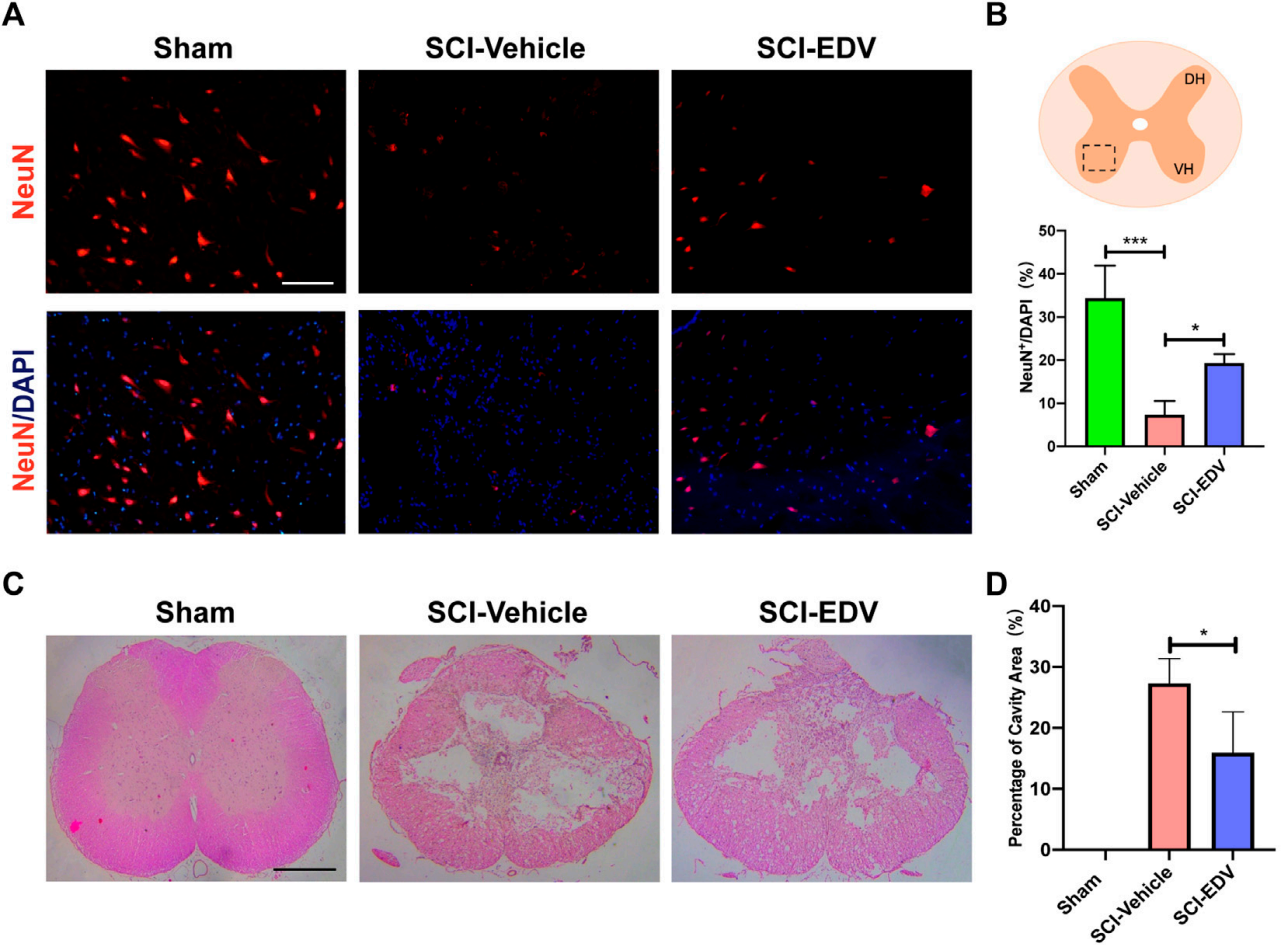
Edaravone has a long-term effect on neuronal survival, reduced astrogliosis, and tissue sparing. **(A)** Representative images of immunofluorescence staining of NeuN in the injury epicenter of 8 weeks post SCI **(B)** Quantifications of NeuN. **(C)** Representative images of H&E staining of spinal cord sections at 4 weeks post injury. **(D)** Quantification of cavity areas made by ImageJ. Data are expressed as mean ± standard deviation. Statistical significance was determined by one-way ANOVA with Tukey’s *post hoc* test. *p < 0.05, and ***p < 0.001, *n* = 3, bar = 100 μm **(A)**, bar = 500 μm **(C)**.

Neuronal survival is the basis of an effective functional circuit. Immunofluorescence staining for NeuN at 8 weeks post SCI decreased significantly after SCI, whereas the treatment of edaravone increased NeuN-positive fluorescence cells, reflecting more neuronal survival (Figures 6A,B). The marked square indicates the area of representative images. Representative images of HE staining of the spinal cord epicenter are shown in Figure 6C. Even after 4 weeks post injury, there is an obvious cavity in the SCI-vehicle group in the lesion epicenter, whereas edaravone treatment spared more tissue and induces a smaller cavity (Figures 6C,D).

### Edaravone Promotes Long-Term Functional Outcome After Spinal Cord Injury

**FIGURE 7:**
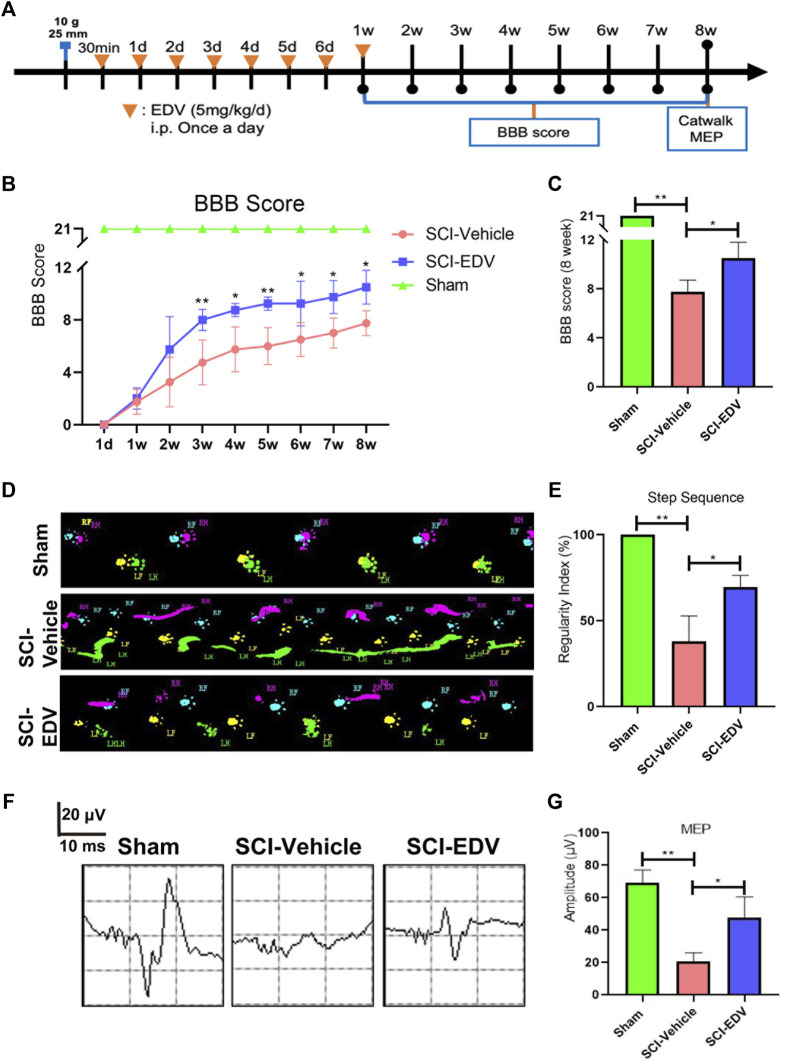
Edaravone promotes motor functional recovery after spinal cord injury. **(A)** Timeline of the experiment. 30 min after SCI, rats received EDV (5 mg/kg/day) continuously for 7 days. BBB scores were assessed every week for 8 weeks. In addition, Catwalk and MEP detection were performed at 8 weeks after SCI. **(B)** BBB locomotor scores were assessed for hindlimbs, up to 8 weeks post injury. **(C)** BBB scores at 8 weeks were compared. **(D)** Print views were recorded at 8 weeks post injury by the CatWalk XT system. **(E)** Regularity index (% index) is presented as an overall measure of the degree of the normal step sequence. **(F)** Electrophysiology of MEP at 8 weeks post spinal cord injury. **(G)** MEP amplitudes of each group were quantified. Data are expressed as the mean ± standard deviation. Statistical significance was determined by one-way ANOVA with Tukey’s *post hoc* test. *p < 0.05; **p < 0.01, *n* = 3.

As a final arbiter of the therapeutic efficacy of edaravone on experimental SCI, assessment of long-term locomotor and electrophysiology outcome is necessary. The functional assessments to confirm the effectiveness of edaravone on SCI are conducted up to 8 weeks post SCI (Figure 7A). BBB score is a standardized locomotor rating scale for open-field testing in rats (Basso et al., 1995). BBB scores were performed weekly till the 8th-week post injury to show the time course of recovery. The treatment of edaravone significantly elevated the BBB locomotor scores compared with those in the SCI group starting from 3 to 8 weeks after injury (Figures 7B,C). Another important assessment in recovery from SCI is the coordination of locomotion (Hamers et al., 2006). Catwalk analysis evaluates the coordination of forelimb and hindlimb. Catwalk-recorded footprint views show improved gait under the treatment of edaravone (Figure 7D). The Regularity Index (RI), a measure of step sequence patterns, as a major coordination parameter, decreased significantly at 8 weeks post SCI and was higher in the SCI-EDV group than in the SCI-vehicle group (Figure 7E). To further evaluate the neurological recovery, the changes of motor-evoked potential (MEP) electrophysiology were applied. The amplitude of MEP returned after edaravone administration (Figures 7F,G). This electrophysiology improvement correlates well with the anatomical and locomotor recovery after SCI.

## Discussion

**FIGURE 8:**
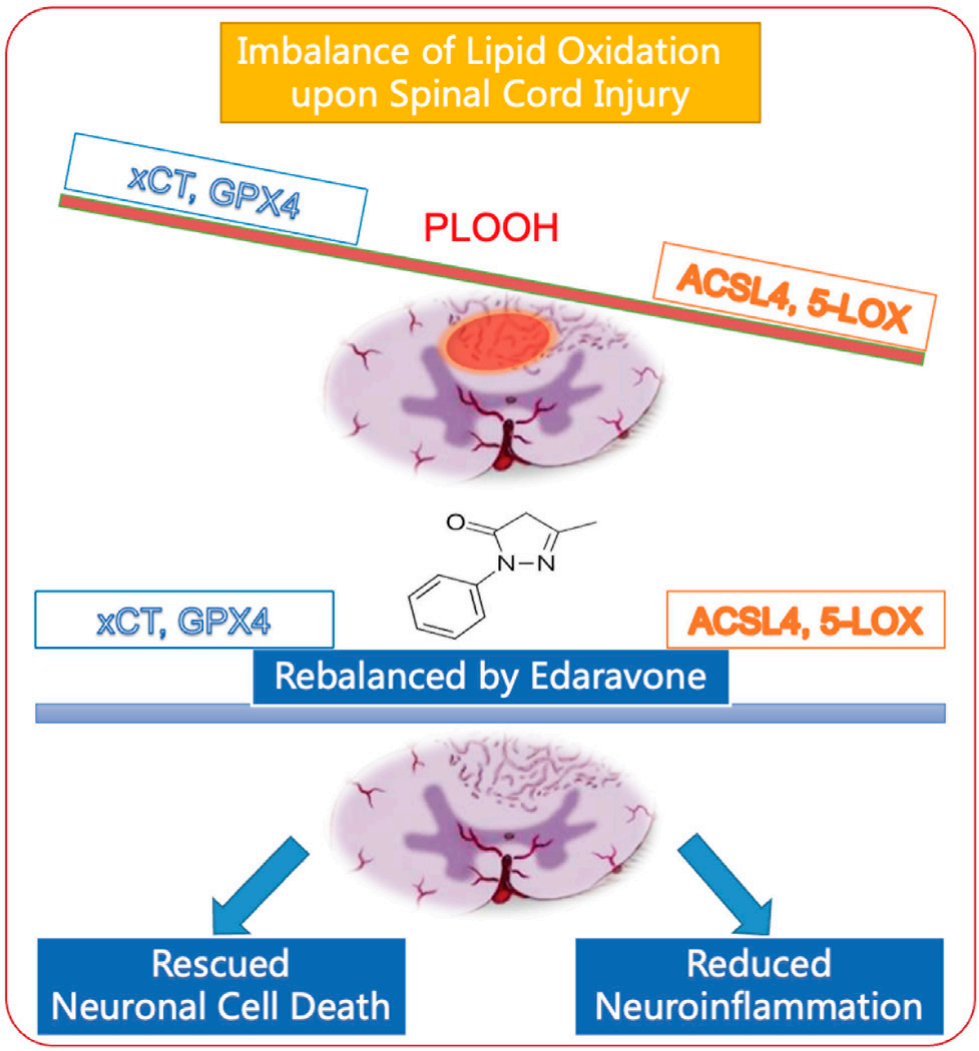
Schematic diagram of edaravone’s neuroprotective mechanism in spinal cord injury. Edaravone upregulates anti-ferroptosis protein GPX4 and xCT and downregulates pro-ferroptosis protein ACSL4 and 5-LOX. This leads to neuronal survival as well as reduced neuroinflammation, and improves recovery of SCI.

While edaravone has proved to have beneficial effect in spinal cord injury, the mechanism is largely unknown. Our study that edaravone inhibit ferroptosis in SCI may be generalizable to the other CNS diseases. In this study, edaravone decreased 5-LOX and ACSL4 expression while increasing GPX4 expression in spinal cord tissue both lesion epicenter and in the caudal lumbar segment. Interestingly, for the first time we also reveal the in neurons, especially those motor neurons in lumbar spinal cord caudal to lesion also undergo ferroptosis and was rescued by edaravone (Figure 8). As a result, edaravone reduces neuronal loss, neuroinflammation, and tissue damage, which aids in long-term locomotion recovery.

In this study, from either the western blot or the immunofluorescence of the spinal cord, we can see edaravone attenuates ferrotoptosis pathway in SCI. We had previously reported that the expression of xCT and GPX4 is downregulated following SCI (Yao et al., 2019; Zhang et al., 2019). xCT is the light chain of SLCA11 (cystine/glutamate antiporter), which imports cysteine and exports glutamine for cells (Dixon et al., 2012). Cysteine is the building block of glutathione, which is also the key to combating ferroptosis. GPX4 is the central regulator of ferroptosis and is responsible for eliminating oxidative damage in cell membranes (Yang et al., 2014; Ingold et al., 2018; Shin et al., 2018). Edaravone rescues both xCT and GPX4 level after SCI. The upregulation of GPX4 is consistent with the recent mechanism revealed of edaravone on depression, and GPX4 knockdown abolished the effect of edaravone treatment (Dang et al., 2022).

Recently the study reported the anti-ferroptosis role of edaravone *via* the anti-ferroptosis proteins, the Nrf/GPX4 pathway on depression (Dang et al., 2022). Here on the other side, we found edaravone reduced the pro-ferroptosis ACSL4 and 5-LOX in SCI. Therefore, this ferroptosis inhibition effect of edaravone are on two sides. The ACSL4 indicates the sensitivity of ferroptosis (Doll et al., 2017). Upregulation of ACSL4 exacerbates the brain injury by promoting ferroptosis (Cui et al., 2021). We found significant incresase in ACSL4 level in neurons, speicifically in motor neurons, indicating the ACSL4 may also act as regulator of neuronal death.

Upon SCI, the 5-LOX level shows significant elvation. 5-LOX may contribute to lipid hydroperoxides, which drives cell from ferroptosis (Shah et al., 2018). And the localization of 5-LOX is mainly in the neuron. This study highlighted the downregulation of 5-LOX by edaravone. Although edaravone is reported to lower 5-LOX in neuronal cells *in vitro* (Song et al., 2018), our study revealed the *in vivo* results that in both the lesion and the lumbar caudal spinal cord, edaravone reduced neuronal 5-LOX, specifically, those in the motor neurons. Mice deficient in 5-LOX display reduced damage following SCI (Genovese et al., 2005), and the 5-LOX inhibitor zileuton improves functional recovery following SCI in rats (Genovese et al., 2008). And ziluton exerts its neuroprotection effect by inhibiting ferroptosis *in vitro* (Liu et al., 2015). 5-LOX affects the arachidonic acid metabolism, so the arachidonic acid metabolites changes in the spinal cord injury is not clear. Therefore, the role of 5-LOX and its relation to ferroptosis in SCI needs further study.

Previous SCI neuroprotection studies mostly focus on the neuronal loss of lesioned spinal cord epicenter. However the nearby segments also suffer from secondary cell death, which hinder recovery. Here we not only look at the epicenter, but also the caudal spinal cord segment. In the caudal lumbar region, the trend of changes in those ferroptosis key proteins are similar as in the epicenter. The neuronal GPX4 decreased dramatically in the caudal part. However, the oligodendrocyte GPX4 changes are not so significant. This difference may be the difference between cell types of the sensitivity of ferroptosis. And edaravone reduced 5-LOX more significantly from the caudal spinal cord than the lesion epicenter. This indicates ferroptosis regulation of neuronal cell death may be expanded from injury site to the nearby spinal cord segments. Here, from the cytokine protein assay screening, we show that edaravone has a marked anti-inflammatory effect on SCI. EDV elevated several anti-inflammatory factors, such as IL-10, IL-13, and adiponectin. IL-10 is known as an anti-inflammatory cytokine in SCI (Thompson et al., 2013). IL-13 is reported to protect against demyelination and improves functional recovery via cell-based delivery after SCI (Dooley et al., 2016). Adiponectin is also an emerging anti-inflammatory factor in SCI. Adiponectin could suppress myelin lipid accumulation and inhibits macrophage recruitment after SCI (Zhou et al., 2019). These anti-inflammatory cytokines increased upon edaravone treatment, indicating ferroptosis is closely related to inflammation, and reversed by edaravone. In addition, edaravone inhibits microgliosis and astrogliosis, further indicating reduced neuroinflammation. Ferroptosis is believed to be related to necroinflammation, especially in neurodegenerative diseases (Proneth and Conrad, 2019). This is consistent with the anti-inflammatory effect of DFO and SRS 16-86 in SCI, where were both ferroptosis inhibitors. The neuroinflammation is also linked with GPX4 reduction since GPX4 conditional knockout mice show increased activity of GFAP (Chen et al., 2015).

## Conclusion

All together, the effect on edaravone has two sides. It not only rescues the ferroptosis negative regulators, xCT and GPX4, but also downregulates those pro-ferroptosis factors, ACSL4 and 5-LOX (Figure 8). In the acute phase of SCI, edaravone reduced neuronal cell death and neuroinflammation. Finally, these acute effects improve long-term functional recovery. Combined with the clear clinical benefit of the drug on CNS diseases, edaravone is a promising pharmacological therapy for SCI. This mechanism is generalizable to other neurological diseases.

## Data Availability

The original contributions presented in the study are included in the article/Supplementary Material, further inquiries can be directed to the corresponding authors.
