# Supplementary figures and images for: Edaravone Modulates Neuronal GPX4/ACSL4/5-LOX to Promote Recovery After Spinal Cord Injury
Source: Front Cell Dev Biol. 2022 May 18;10:849854. doi: 10.3389/fcell.2022.849854 (PMC9318422; doi:10.3389/fcell.2022.849854)

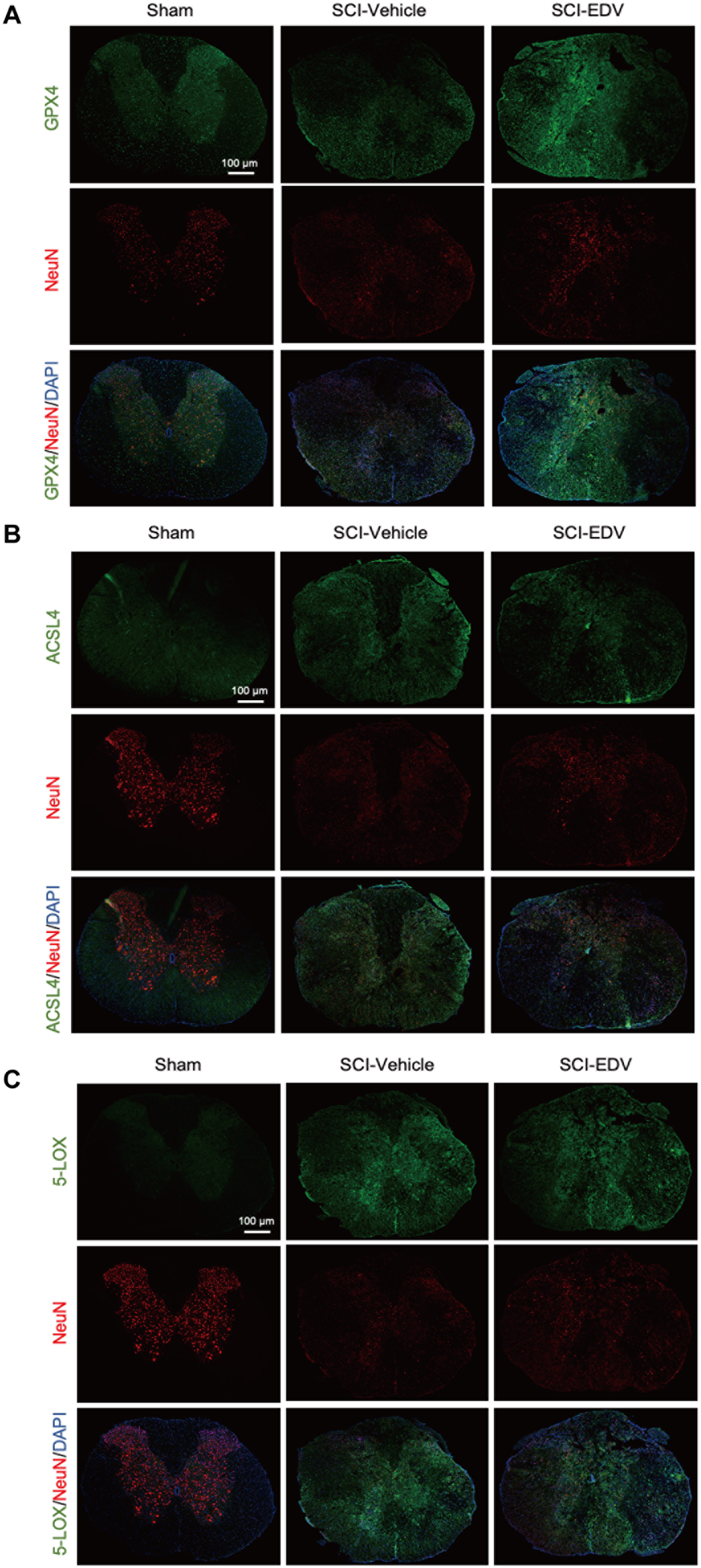

Supplement: Supplementary file 1 [file Image2.tif]

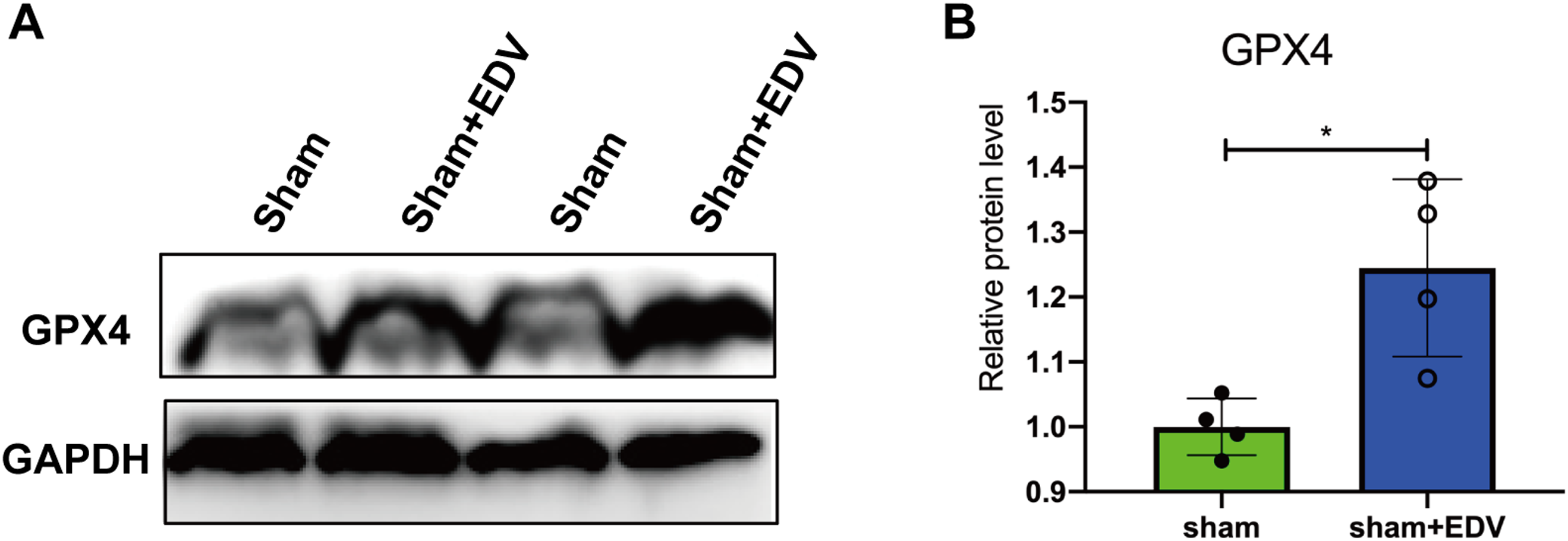

Supplement: Supplementary file 2 [file Image1.tif]
